# Fabrication of chicken 3D-analogs *via* soy proteins hydrocolloid-mediated noncovalent complexation with lipo-ligands: Mechanistic elucidation and structure-function dynamics

**DOI:** 10.1016/j.fochx.2025.102664

**Published:** 2025-06-13

**Authors:** Remah Sobhy, Ke Zhang, Jianing Zhang, Mohammed Alrugaibah, Thamer Aljutaily, Raed Alayouni, Hassan Barakat, Ibrahim Khalifa, Xiaobo Zou

**Affiliations:** a Agricultural Product Processing and Storage Lab, School of Food and Biological Engineering, Jiangsu University Zhenjiang, Jiangsu 212013, China.; b Department of Food Science and Human Nutrition, College of Agriculture and Food, Qassim University, Buraydah 51452, Saudi Arabia.

**Keywords:** Lipophilic phytonutrients, Food supply, Mechanism, Protein-ligand binding, 3D-chicken analogs

## Abstract

We aimed to develop 3D-printed chicken analogs with improved structural integrity by leveraging soy protein interactions with lipo-ligands, stabilized by gellan gums. We thus fabricated four formulations and systematically evaluated their rheological, textural, thermal, and structural properties. Additionally, we used molecular docking models to elucidate binding affinities among ingredients. Results indicated that protein-ligand interactions, particularly those involving γ-oryzanol and gellan gum, significantly improved printability, and structural stability. Multispectral and molecular docking analyses revealed that soy protein-oryzanol complexes synergistically reinforced noncovalent interactions (H-bonding and hydrophobic) with gellan gum, enhancing ink cohesion and water retention. These formulations demonstrated optimal viscoelasticity for extrusion and maintained shape fidelity after printing. Thermal analysis confirmed that protein-oryzanol complexes exhibited greater stability and stronger 3D-printed layer adhesion compared to single-proteins. These findings underscore importance of tailored protein-multi-lipo-ligands in plant-based meat, with soy proteins-oryzanol systems offering a promising route to structurally coherent chicken-analogs for sustainable 3D-food printing applications.

## Highlights

• SPI-Ory-GG synergy enables superior 3D-printing of plant-based chicken analogs.
• This method improves ink cohesion, water mobility, and extrusion performance.
• SPI-Ory-GG boosts thermal stability and layer adhesion in printed products.
• Tailored SPI-lipo-ligands advance sustainable, realistic chicken alternatives.
• We innovatively employed computational chemistry in designing plant chicken analogs.

## 1 Introduction

The escalating global demand for plant-based meat analogs stems from mounting environmental, ethical, and health concerns, prompting both industry and academia to seek sustainable alternatives that can satisfy consumer expectations for taste, texture, and nutrition (Rout & Srivastav, 2024). Among these, chicken analogs have gained particular attention due to chicken's ubiquity in global diets and its characteristic fibrous, juicy texture, which remains challenging to replicate using plant proteins alone (De Angelis, van der Goot, Pasqualone, & Summo., 2024). Although extrusion and shear-cell processing have advanced the field, these methods often fail to reproduce the anisotropic muscle structure and succulence of real chicken, typically yielding products with spongy or uniform textures (Kyriakopoulou et al., 2019). Hybrid meat analogs, which combine plant proteins with animal-derived ingredients, offer a partial solution but do not fully align with efforts to minimize animal inputs and achieve authentic meat-like properties (Dekkers & Jan Van Der Goot, 2018). Recent advances in 3D food printing enable fabrication of complex, layered structures that better mimic muscle tissue architecture. However, the rheological and binding properties of current protein matrices limit print resolution and product stability (Chen et al., 2024). Overcoming these technical barriers requires a deeper molecular understanding of how plant proteins interact with functional additives to enhance viscoelasticity and structural cohesion. Therefore, this study aims to elucidate the molecular mechanisms underlying protein-additive interactions in plant-based matrices and to identify strategies for optimizing the textural fidelity and functional performance of chicken analogs, contributing to the development of next-generation meat alternatives that are both appealing and sustainable.

Among different plant protein sources, soy protein isolates (SPI), particularly glycinin (11S) and β-conglycinin (7S) exhibit versatile binding capacities with lipophilic phytonutrients (Li et al., 2024), mainly governed by spontaneous hydrophobic interactions, H-bonding, and electrostatic forces. For example, SPI-lutein (Wang, Wang, Xu, Peng, Gao, & Cao, 2022) and SPI-astaxanthin (Ge et al., 2024) formed stable complex *via* hydrophobic forces and H-bonding (C=O/amine). These interactions increase the bioavailability of lipophilic phytonutrients and alter protein structures, serving as both structural modifiers and nutraceutical carriers. Yet competitive binding dynamics between protein subunits and phytonutrients under 3D-printing shear stresses remain poorly understood. Current methods fail to monitor these interactions in real time during printing, overlooking critical shear-thinning and rapid gelation effects. Bridging this gap requires integrating *in situ* spectroscopy with computational modeling to predictively design tailored protein–lipo-ligand matrices for additive manufacturing.

Harnessing molecular interactions enables innovative engineering of chicken analogs with aligned fibers and moisture retention. Adjusting SPI-lipo-ligand ratios, tailors bio-ink viscoelasticity for extrusion printing (Lee, Oh, An, Kim, & Kim, 2020). Lutein's planar structure boosts SPI β-sheets, aiding fibril formation; astaxanthin's conjugated dienes stabilize disulfide bridges, preventing delamination; and γ-oryzanol's amphiphilic nature acts as a plasticizer, reducing clogging while enhancing cohesion *via* hydrophobic cross-links. These principles align with food science's “structure-by-design” paradigm, positioning lipo-ligands as dual-purpose structural and nutritional enhancers in next-gen meat analogs. Based on these findings, we hypothesized that the structural diversity of lutein, astaxanthin, and γ-oryzanol would differentially modulate their conjugation with SPI, thereby altering the formulation of plant-based chicken analogs. Thus, this research investigated the binding interactions between lutein, astaxanthin, and γ-oryzanol with SPI from molecular modeling to experimental validation to develop 3D-printed chicken analogs. The conjugation between lipophilic pigments and proteins was first fluorometrically and analyzed *in-silico*. Second, the formulated lipo-ligand-protein analogs were examined for their appearance, rheological, textural, and microstructural properties.

## 2 Materials and methods

### 2.1 Materials

SPI (91.5 % protein by dry weight, with all particle sizes passing 20 mesh, 2.5 % ash) were sourced from Xi'an Green Spring Technology Co., Ltd., Xi'an, China. High acyl gellan gum (99 % pure, 80mesh ≥ 98, E418) was obtained from Zhengzhou Cinogel Biotech Co., Ltd., Zhejiang, China. Lutein (CAS No.: 127–40-2, HPLC ≥98 %), astaxanthin (CAS No.: 472–61-7, HPLC ≥90 %), and γ-oryzanol (CAS No.: 11042–64-1, HPLC ≥98 %) were acquired from Shyuanye Co., Ltd., China. Nile red and fluorescein isothiocyanate (FITC) were purchased from Sigma-Aldrich. All other chemicals and kits were of analytical grade, and MillQ-H_2_O was utilized throughout the investigation.

### 2.2 Food ink formulation and 3D-printing setup

SPI powder was weighed to achieve a final concentration of 18 wt% and initially blended in a laboratory mixer for 5 min at room temperature to ensure uniform dispersion. Subsequently, MilliQ-grade deionized water was gradually incorporated into the mixture and stirred continuously for 20 min at 25 °C to form a consistent, hydrated base. After hydration, gellan gum (GG) was added at 2 wt% and thoroughly mixed into the blend. Depending on the experimental group, either lutein (Leu, 0.5 wt%), astaxanthin (Ast, 0.5 wt%), or γ-oryzanol (Ory, 0.5 wt%) was introduced individually, and the mixture was kneaded for an additional 30 min until a macroscopically homogeneous food ink was achieved.

The prepared food inks were loaded into a 10 mL syringe fitted with a conical needle (200 μm inner diameter; 600 μm was also tested for comparison) and mounted onto a custom-built 3D syringe pump extrusion system. This system featured a computer-controlled three-axis positioning stage (work area: 45 × 40 × 40 cm^3^), enabling precise movement of both platform and nozzle along the x, y, and z axes. During 3D printing, the following parameters were strictly maintained: nozzle diameter (200 or 600 μm), extrusion rate (32.5 mL/min), XY-axis traversal speed (1350 mm/min), protein concentration (18 wt%), individual additive concentrations (Leu, Ast, or Ory at 0.5 wt% each), and GG content (2 wt%). All samples were printed under these fixed conditions to ensure consistency across experiments.

### 2.3 Measuring the mechanism of protein-lipo-ligands binding gum-stabilized in chicken analogs

#### 2.3.1 Multispectral experimental binding measurements

A UV-8000 spectrophotometer (Shanghai, China) was used to examine the inks' ultraviolet (UV) absorption characteristics. To prepare the samples, the inks were diluted at a 1:3 (*v*/v) ratio in PBS (pH 7.4) and then vortexed for 10 min at 600 rpm. The wavelength range of the measured absorption spectra was 200–600 nm. Fluorescence intensity was measured concurrently. Ink solutions (5 mg mL^−1^) in NaCl at pH 7 were agitated for 3 h at 25 ± 3 °C to perform fluorescence analysis. A Cary Eclipse fluorescence spectrophotometer (Varian, Australia) was used to get the fluorescence emission spectrum. With an excitation wavelength of 280 nm and excitation and emission slit widths of 2.5 nm each, emission spectra were obtained between 300 and 450 nm. Each ink's dominant emission peaks were located, and the maximum fluorescence peak quenching values were measured (Khalifa et al., 2020).

FTIR Nicolet 470 (Thermo Fisher Scientific, US) was used to capture the FTIR spectra for each lyophilized ink using a KBr disc technique. With a resolution of ±2 cm^−1^ and 21 scans per min, the spectra were recorded in transmission mode between 4000 and 400 cm^−1^. The Omnic program utilized Fourier self-deconvolution and automatically corrected the baselines of the FTIR spectra. Since the amid-I band of the peptide backbone is anticipated to be absorbed in this area, the spectral range between 1600 and 1700 cm^−1^ was chosen to investigate the modification that happened. PeakFit software version 4.12 (SPSS Inc., Chicago, IL, US) was used to do the second derivative computations and a multiple Gaussian curve-fitting analysis to estimate the number and location of the component bands (Khalifa, Peng, et al., 2019).

#### 2.3.2 Molecular docking theoretical modeling and dynamic simulation

The bottom-up design framework was started by molecular docking, which made it possible to evaluate and characterize the binding affinities among the components of chicken analogs. The RCSB (https://www.rcsb.org) and PubChem (https://pubchem.ncbi.nlm.nih.gov) databases provided the crystal structures of glycinin A3B4 subunit homohexamer (SPI's core protein; PDB:1OD5), Leu (PubChem-CID: 5281243), Ory (PubChem-CID: 5282164), Ast (PubChem-CID: 5281224), and GG (PubChem-SID: 505635529). Our 3D-printed chicken analogs were designed using glycinin as the receptor, which was sequentially/combinatorially docked with each lipo-ligand individually to produce SPI-lipo-ligand conjugates. To display the protein-protein-multiple-ligand interaction, each SPI-lipo-ligand was also individually docked with gellan Gum to produce SPI- lipo-ligand-GG complexes. Meanwhile, each lipo-ligand and/or GG were considered ligand-protein. Using protonate-3D and the MMFF94X force field, structures were optimized by charge integration and energy reduction. After removing the water molecules from glycinin and refining its shape using Amber10: EHT forcefield (0.1 RMS kcal/mol/Å^2^), 3D protonation was performed in Discovery Studio, version 2.5. Site-finder techniques were used to identify the binding sites for either lipo-ligand or carbohydrate-based ligands, and fake atoms were placed in the biggest cavity. London dG scoring and forcefield algorithms were used to produce five ideal docking conformations. Surface mapping and particle mesh Ewald approaches were used to quantify long-range hydrophobic and electrostatic interactions (Khalifa et al., 2023). Heatmaps were used to depict patterns of interaction based on Pearson correlation coefficients (http://heatmapper.ca/expression) and visualized interaction patterns. The specific protein-ligand interactions were identified and characterized using the Protein-Ligand Interaction Profiler (PLIP) server (https://plip-tool.biotec.tu-dresden.de/plip-web/plip/index).

### 2.4 Evaluating the printability of SPI-lipo-ligands gum-stabilized edible inks

First, the edible inks were evaluated using low-field nuclear magnetic resonance (LF-NMR) (Mohammed et al., 2025). Using a nuclear magnetic resonance analyzer (NMI20–015 V–I, Niumag Co., Ltd., China) fitted with a 0.5 T permanent magnet, which yielded a proton resonance frequency of 21.16 MHz at 32 °C, the spin-spin relaxation durations (T_2_) for the food inks were measured. Before being inserted into the analyzer, each 20 g food ink was wrapped in a thin layer of plastic film and put inside a 40 mm glass tube. Using the following settings, the T_2_-relaxation duration was determined using Carr-Purcell-Meiboom-Gill sequences: Duration waiting (TW) = 500 ms, echo time (TE) = 0.17 ms, number of echoes (NECH) = 5000, and number of scans (NS) = 4. Using the program MultiExp Inv Analysis 4.0909 (Niumag Electric Co., Ltd., China), the data were examined using both multi-exponential and mono-exponential fitting of T_2_-relaxation data.

Second, the food inks were rheologically evaluated using a rheometer (MCR102, Anton Paar, Austria) equipped with a parallel plate of 25 mm in diameter. To verify the shear thinning properties of food inks, viscosity was assessed by varying the shear rate from 1 to 500 s^−1^. The dynamic frequency sweep test examines the flow characteristics during the extrusion of food ink from the syringe nozzle during printing. Herein, the storage modulus (G') and loss modulus (G") were measured at a fixed strain of 2 % while varying the frequency over a range of 0.1–100 rad/s. G′ and G″ were measured at a steady frequency of 10 s^−1^ while changing the strain from 0.01 to 1000 % (Jia et al., 2022, Zhao et al., 2022).

### 2.5 Characterizing the 3D-printed chicken analogs

#### 2.5.1 Texture profile analysis (TPA)

Using a Texture Analyzer (CT3-Texture Analyzer, Brookfield, Middleboro, MA) and a two-consecutive-cycle compression approach, the textural characteristics of 3D-printed chicken analogs were assessed (Khati, Le Parc, Chevalier-Lucia, & Picart-Palmade, 2024). A 50 mm stainless steel cylinder probe was used to investigate the analogs after they were cut into uniform 10 mm cubic specimens. The settings that were sat as follows: 50 % maximum deformation, 15 g automated trigger force, 5 s between compressions, and constant pre-, intra-, and post-test speeds of 2 mm s^−1^. In accordance with a defined procedure, key parameters such as chewiness, resilience, hardness, and springiness were computed to quantify structural properties (Lu et al., 2022).

#### 2.5.2 Visual appearance and microstructure

After being frozen for 12 h at −80 °C, each 3D-printed chicken analog was then freeze-dried. To guarantee cross-sectional visibility, the gel cross-sections were physically broken to reveal them and then adhered to a sample stage using conductive glue. Scanning electron microscopy (SEM; Gemini300, Zeiss Int, Germany) operating at 2 kV was used to perform microstructural investigation after gold sputter coating. Simultaneously, analog microstructures were seen using fluorescent microscopy (Nikon Eclipse Ti—S, Nikon Instruments Inc., US) using 20× and 40× lenses. This was accomplished by combining 2 mL of protein extracts from each analog, as previously described (Khalifa, Peng, et al., 2019), with 40 μL of 0.1 % FITC (in DMSO; λex/λem = 494/518 nm), incubating for 1 min at 25 °C, and then imaging. A Nikon DS-Fi 2.5 camera (Japan) was used to take the micrographs, and NIS-Elements software (v4.4, Nikon, Japan) was used to analyze the images (Taha et al., 2018).

#### 2.5.3 Thermal stability

We used a differential scanning calorimeter (DSC) (VP-Capillary-DSC Q20, Thermal Analysis Corp., DE, US) to measure the thermal denaturation temperatures of the analogs (Khalifa, Li, et al., 2019). We selected the DSC parameters based on a combination of preliminary trials and established practices in food protein analysis to ensure reliable detection of thermal denaturation in the analogs. Specifically, we equilibrated samples at 20 °C to ensure thermal uniformity, then heated them to 110 °C at 5 °C/min-a rate chosen because it balances resolution and sensitivity while minimizing baseline noise and sample degradation, as confirmed by pre-experiments comparing rates between 2 and 10 °C/min. The isothermal hold at 110 °C for 30 min was included to allow complete denaturation and to capture any slower thermal transitions, as observed in initial test runs. The cooling and reheating steps at the same rate were implemented to assess the reversibility of denaturation and ensure consistency in thermal profiles. All DSC pans were sealed hermetically to prevent moisture loss, and an empty pan served as a reference. The integrated software of the device was used to determine denaturation temperatures (Tm) and heat flow profiles. These conditions were validated in preliminary experiments to optimize signal clarity and reproducibility for our specific analog matrix, ensuring the robustness of our thermal analysis.

In parallel, X-ray diffraction (Malvern Panalytical XPert Pro MPD) was used to analyze the analogues' crystalline and amorphous phases. 50 mg of lyophilized analogs were scanned at a rate of 4°/min from 5° to 90° while operating at 40 kV and 40 mA.

### 2.6 Statistical analysis

The results were expressed as the mean value ± SD, and each test was conducted in triplicate. To compare the mean values at a significance level of *p* < 0.05, statistical analysis was conducted using SPSS 27.0 (SPSS Inc., CHI, US), which included one-way analysis of variance (ANOVA) with Duncan's post-hoc test. To illustrate and display the data, OriginPro 2025's educational edition with Jiangsu University accessibility was utilized.

## 3 Results and discussion

**Fig. 1:**
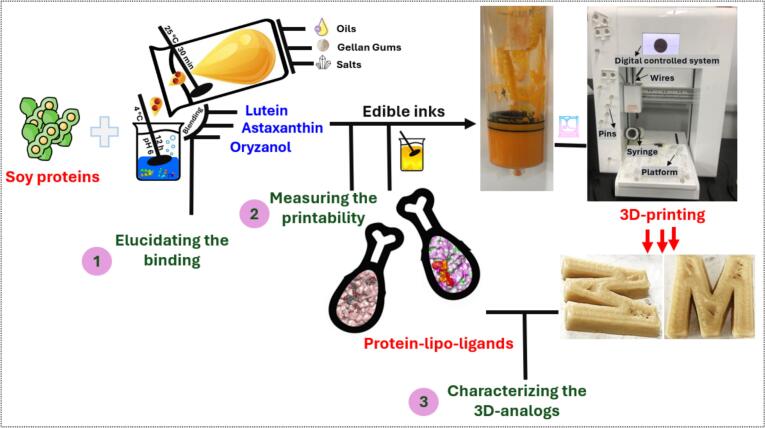
Schematic of the 3D-printing system and the detailed steps of formulating 3D-printed chicken analogs based on protein-lipo-ligands interaction stabilized by Gellan gums and their analysis.

Fig. 1 illustrates the development schematic of plant-based chicken analogs incorporating SPI interactions with lipophilic bioactive components such as Ast, Ory, and Leu, stabilized by GG within a 3D-printing platform. The system employs a digitally controlled extrusion setup comprising syringes, pins, and a temperature-regulated platform to optimize layer-by-layer deposition. Four formulations were prepared by systematically adding each lipo-ligand, while the control sample was only SPI, and GG was presented in all samples with a fixed concentration. GG facilitated oil-in-water emulsion stability, while protein-lipo-ligand interactions were modulated to enhance printability and structural fidelity. These blends were processed through a high-moisture extrusion system, leveraging temperature, pressure, and shear forces to induce protein alignment and cross-linking.

### 3.1 Lipo-ligands effectively interacted with soy proteins in the presence of gellan gums in ink models

**Fig. 2:**
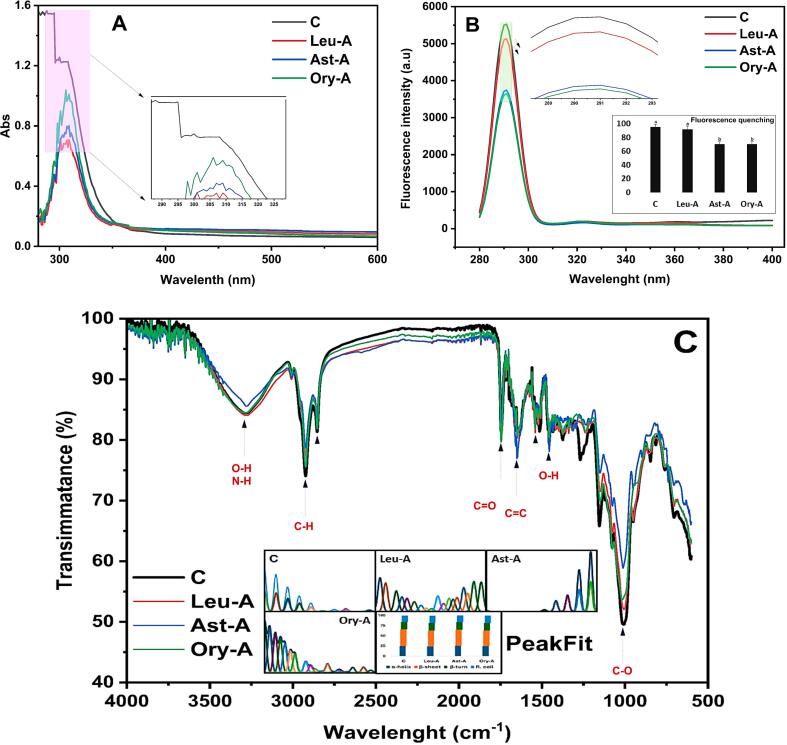
The UV–vis spectroscopy (A), fluorescent intensity and quenching (B), and FTIR peaks, as well as peakfit analysis with their secondary structure analysis (C) of each chicken analog-based ink prepared from SPI with three lipo-lipid ligands.

The integration of SPI with lipophilic phytonutrients (Ast, Ory, and Leu) in the presence of GG as a stabilizer was systematically investigated to elucidate structural and functional synergies critical for chicken analog development (Fig. 2). Such multispectral data provides comprehensive insights into protein-lipo-ligand-gum interactions in terms of elucidating the mechanism beyond their different printability behavior. The UV–Visible spectroscopy revealed distinct absorbance peaks at ∼280–300 nm (Fig. 2 **A**), indicative of aromatic amino acid residues in SPI and their interactions with phytonutrients (Zhao et al., 2022). Meanwhile, SPI showed a higher absorbance intensity, whereas formulation containing Leu exhibited lessened absorbance (∼0.7 AU), suggesting π-π stacking or hydrophobic interactions that enhance complex stability. Meanwhile, each of Ast and Ory diminished the intensity of the UV–visible peak of SPI, where both also fluctuated and shifted the SPI peak at ∼280–300 nm, suggesting that each lipo-ligand had the ability to interact with SPI, thereby differently moderates its overall aromatic amino acid exposure. Protein-ligand interactions are strongly supported by the red and/or blue color changes in absorbance maxima of SPI that have been seen with different lipo-ligands. Previously, Tian et al. (2023) and Li et al. (2024) showed that β-carotene and/or astaxanthin could interact with SPI-fibrils in plant-based models. Fig. 2**B** showed the fluorescence spectroscopy of the binding between SPI-lipo-ligands in the presence of GG in edible ink models. The emission spectra (280–400 nm) display characteristic peaks at ∼285–295 nm, indicative of intrinsic Try and Tyr fluorescence from SPI's aromatic amino acid residues. The four samples demonstrate differential quenching effects, with the inset bar graph quantifying this phenomenon. SPI and SPI-Leu samples exhibit similar high fluorescence intensity, while SPI-Ast and SPI-Ory show significant quenching (∼30 % reduction), indicating stronger binding interactions with SPI. This fluorescence quenching pattern represents a static binding mechanism between SPI and bioactive compounds, consistent with the Stern-Volmer relationship used to characterize protein-ligand interactions.

The fluorescence spectroscopy of SPI-lipo-ligand binding in the presence of GG in edible ink models was displayed in Fig. 2**B**. The distinctive peaks at ∼285–295 nm in the emission spectra (280–400 nm) show intrinsic Try and Tyr fluorescence from the aromatic amino acid residues of SPI. While SPI-Ast and SPI-Ory display considerable quenching (∼30 % decrease), suggesting greater binding interactions with SPI, SPI and SPI-Leu samples show similar high fluorescence intensity. According to the Stern-Volmer relationship, which is used to describe protein-ligand interactions, this fluorescence quenching pattern indicates a static binding mechanism between SPI and bioactive substances (Khalifa et al., 2022). In contrast to Leu and/or Ast, the differential quenching indicates that Ory creates more persistent hydrophobic contact with SPI. Conformational changes in SPI-structure upon bioactive binding are further confirmed by the observed red shift in the emission spectrum, which can be seen in the tiny inset graph. These protein-bioactive binding properties significantly impacted the formulations of chicken analogs. The introduction of GG as a stabilizer improves texture development and emulsion stability through greater SPI-bioactive interactions (Ma et al., 2025). These substances would be more successfully maintained during processing and more stably integrated into the protein matrix, based on the greater binding shown with Ast and Ory. According to recent research using SPI-hydrocolloid systems, improving these binding interactions has a direct impact on the mechanical and sensory qualities that are essential for meat substitutes to be effective (Funami et al., 2023). When used in GG-stabilized systems, this differential binding pattern presents a substantial opportunity to enhance the nutritional value and technical performance of plant-based chicken substitutes.

The FTIR spectra presented in Fig. 2**C** reveal significant structural insights into the interactions between SPI and lipophilic bioactive compounds in chicken analog formulations. The spectra display characteristic bands in the amide I (1700–1600 cm^−1^) and amide II (1600–1500 cm^−1^) regions, which are essential for protein secondary structure determination and related to N—H bending coupled with C—N stretching (Chiang et al., 2023). Comparing the four samples (C, Leu-A, Ast-A, and Ory-A), notable variations appear in the peak intensities and slight shifts in wavelengths, particularly in the regions marked with triangles. The PeakFit analysis indicates differential changes in protein secondary structures upon binding with the bioactive compounds (Guerrero, Kerry, & de la Caba, 2014), with the most pronounced differences observed in SPI-Ast and SPI-Ory samples. SPI-Ory shows distinctive peaks in the 1500–1000 cm^−1^ region, which may correspond to interactions between the ferulic acid moiety of oryzanol and SPI-functional groups. Notably, the broadband at 3300–3500 cm^−1^, corresponding to O—H and N—H stretching vibrations, shows considerable differences in intensity and shape among the inks. SPI-Ory demonstrates the most pronounced dip in this region, indicating enhanced H-bonding networks that are critical for structural integrity in plant-based chicken analogs. The 1000–1200 cm^−1^ range, which is linked to C—O stretching vibrations, shows structural differences in GG in the presence of SPI. Based on the peakFit analysis, Ory and Ast appear to induce greater β-sheet formation compared to SPI and SPI-Leu, like what Li et al. (2025) observed with rosmarinic acid binding to SPI, where β-sheet content increased by 9.61 % while α-helix content decreased slightly. Because bioactive compounds change protein conformation, they affect water-binding capacity, textural qualities, and fibrous structure formation during high-moisture extrusion. According to these binding interactions, adding these bioactive ingredients and GG-stabilization may improve the nutritional content and structural soundness of soy-based chicken substitutes.

**Fig. 3:**
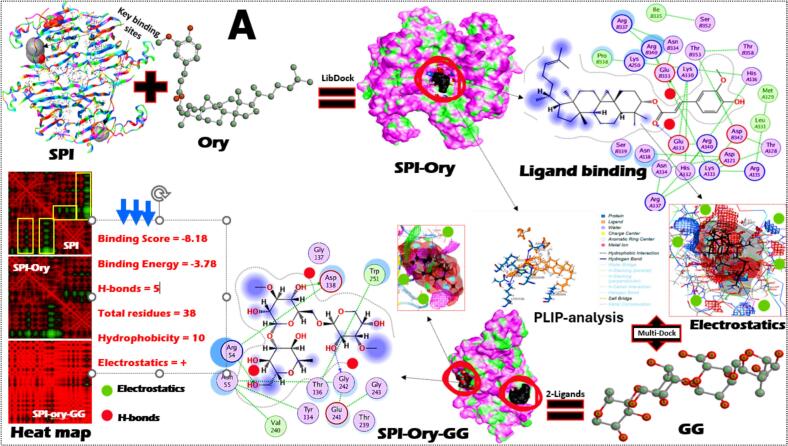

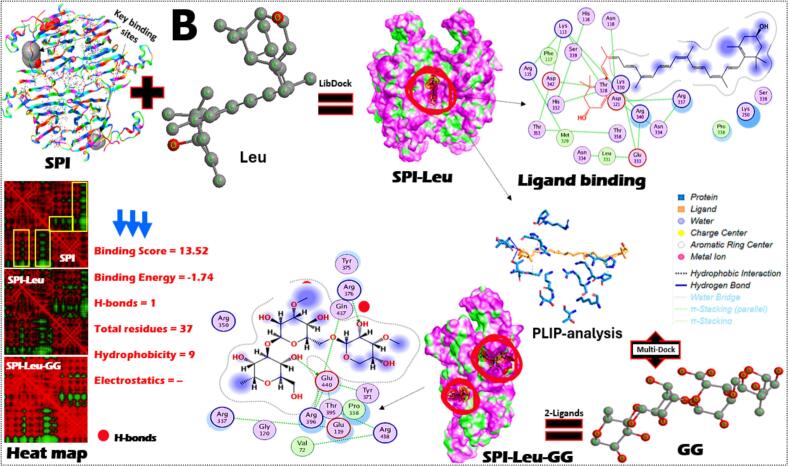

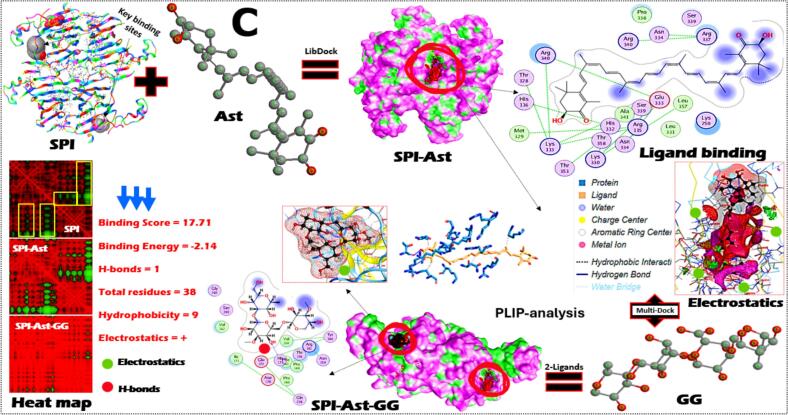
The homology model and key binding sites of soybean proteins (SPI) and their interaction with γ-oryzanol (A), lutein (B), and astaxanthin (C) and their homology structure models, 2D-ligand binding, surface interaction analysis, SPI-lipo-ligand binding with Gellan gum (GG). The PLIP-analysis and heat map analysis of SPI-lipo-ligand-GG conjugates were also presented. The binding constants and experimental binding forces were also stated.

To determine their potential for creating ideal chicken analog formulations, the molecular interactions and binding characteristics of SPI interacted with three lipo-ligands in the presence of GG were examined. Using molecular docking, binding analysis, and dynamic simulations, the protein-multiple-ligand interactions, structural features, and binding affinities were assessed separately as well as in different combinations (Figs. 3A-C). For example, Ory, a mixture of ferulic acid esters and phytosterols found in rice, demonstrates significant binding affinity with SPI (Fig. 3A). PLIP-analysis and heat map indicate that H-bonding is the primary interaction mechanism, with specific amino acid residues (GluB333 and GluA333) in the protein's binding pocket forming stable connections with OH-groups of Ory. H-bonds were also noted after interacting with GG, where Gly242, Asp138, and Arg54 of SPI were involved. The binding constants presented suggest spontaneous interaction (score of −818 and energy of −3.78) with moderate binding strength, which is crucial for maintaining structural integrity. Both electrostatic and hydrophobicity forces were also excited in SPI-Ory-GG binding.

On the other hand, Leu interacts primarily through hydrophobic interactions (9 from a total of 37 residues) with the residues of SPI, with an absence of any H-bonds that might affect this conjugate further stability (Fig. 3B). The only H-bond was found after interacting SPI-Leu with GG, where Arg376 of SPI involved, where the electrostatic forces were absent. The surface interaction analysis reveals that Leu is partially embedded within hydrophobic pockets of the SPI structure. The binding constants shown in the figure correlate with recent studies demonstrating that SPI-Leu form spontaneously through hydrophobic interactions with a binding stoichiometry of 1:1 (Wang, Wang, Xu, Peng, Gao, Cao, & i. N., 2022). Leu binding to proteins often involves one of the ionone rings protruding outside the binding cavity, while the polyene chain extends through the hydrophobic pocket (Qi et al., 2021). Likewise, the 2D-ligand binding analysis reveals multiple interaction points along the extended polyene chain of Ast, forming a network of hydrophobic interactions (9) and electrostatic forces (+) with SPI (Fig. 3C). Meanwhile, one H-bond was noted after integrating GG, where His173 of SPI interacted with OH-groups of GG gums. The binding score and energy values were 17.71 and − 2.14, where the total residues involved in this binding were about 38. This analysis may order the binding of those three lipo-lipids with SPI, where Ory (4H-bonds, 10 hydrophobic and electrostatic forces) was peaked, followed by Ast (1H-bonds, 9 hydrophobic and electrostatic forces) and later Leu (1H-bonds and 9 hydrophobic forces).

Most importantly, the molecular interactions between GG and the SPI-lipo-lipid complexes create a secondary network of stabilizing forces that enhance the overall structural integrity. This polysaccharide-protein-ligand tertiary system provides improved emulsification properties, critical for mimicking the fibrous texture characteristic of chicken meat especially effectively preventing aggregation and phase separation during processing and storage. These properties, combined with its stable binding to SPI, make Ory an excellent candidate for enhancing the nutritional profile and shelf stability of chicken analogs. The green regions in the heat maps indicate strong interaction zones, while the red regions represent weaker interactions. Taken together from the experimental and theoretical results, SPI-lipo-ligands form stable complexes through different binding mechanisms, with Ory showing the strongest binding affinity. The incorporation of GG provides additional stabilization essential for maintaining structural integrity during processing. These interactions are fundamental to developing improved chicken analogs with enhanced functionality and stability profiles.

### 3.2 Printability of protein-protein-multiple-ligands edible inks

**Fig. 4:**
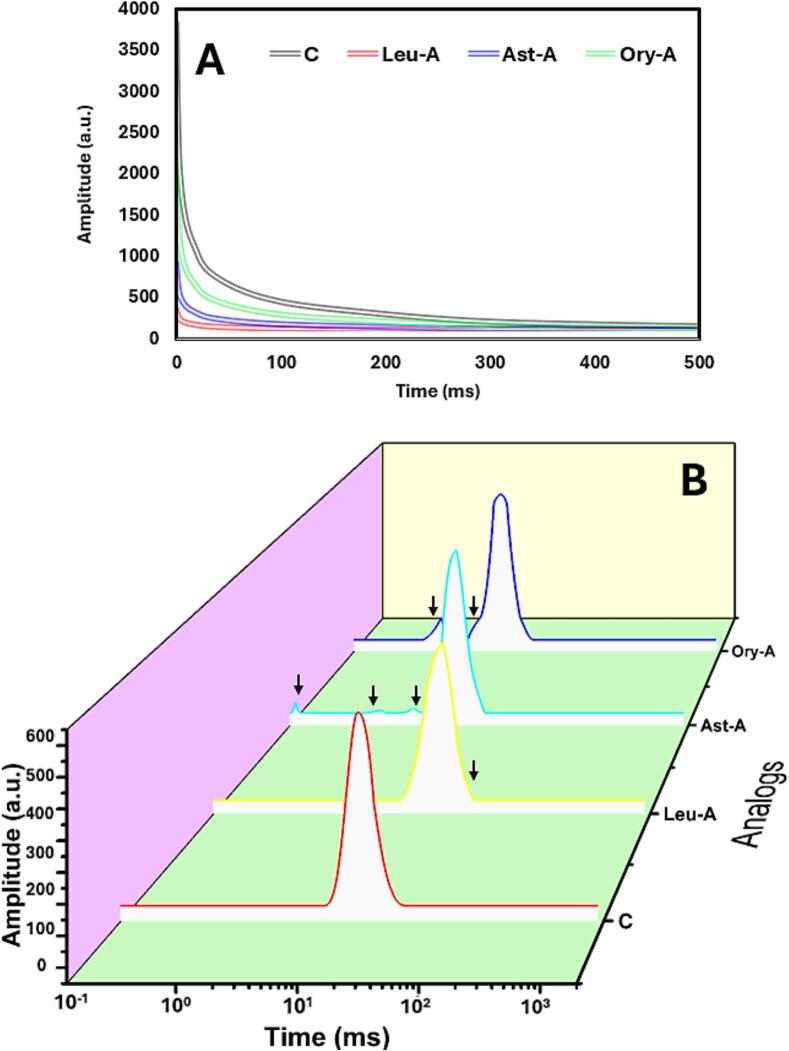
The transverse relaxation behaviors and chemical binding of each chicken analog composite food ink are based on the amalgamations of different formulas, namely relaxation decay curve (A) and distribution of transverse relaxation time (T_2_) spectra (B).

Imperative information on water mobility and molecular interactions in SPI-based inks may be gleaned from the transverse relaxation characteristics (Figs. 4A-B). The distinct protein-ligand matrix architectures and water-binding capacities of each of the four inks are reflected in their distinctive NMR relaxation patterns, which have a direct impact on their technical qualities for use in food applications. The relaxation curves in Fig. 4A depict the four inks' different water mobility patterns. SPI-only has the largest initial amplitude and steepest decline curve, suggesting comparatively stronger water mobility than the other inks (Guo et al., 2024). SPI-Leu, on the other hand, has a greater water-binding capacity and more constrained water mobility, as seen by its significantly smaller initial amplitude and slower decline. With a moderate starting amplitude and decay rate, SPI-Ory/Ast has intermediate water mobility properties. Interestingly, the binding between different lipo-ligands and SPI reveals how such ligands modify water-binding behavior. Among all combinations, SPI-Ory exhibits the most gradual decay profile, indicating this combination most effectively restricts water mobility through stronger binding forces between water and the protein matrix (Tarahi et al., 2022). The distribution of transverse relaxation durations (T₂) for each ink is shown in Fig. 4B, where unique peaks correspond to various water populations in the systems. The arrows draw attention to certain relaxation elements that define each formulation's water states. SPI-only displays the longest T₂ relaxation time among single-protein formulations, indicating SPI creates a matrix that distinctly affects water mobility (Bugday, Venkatachalam, Anderson, & van der Sman., 2024). SPI interaction with the three ligands shows shifted relaxation time distributions, demonstrating that protein-multi-ligands create complex matrices that affect water mobility differently than single-protein systems. Notably, SPI-Ory displays unique distribution patterns, suggesting SPI and Ory combination creates a noncovalent conjugate that affects the water binding and mobility. Regarding its potential applications as edible inks for chicken analogs, SPI might produce products with insufficient structural integrity due to high water mobility, whereas both SPI-Ory and SPI-Ast offer balanced water mobility characteristics that could better mimic the juiciness and texture of chicken. Formulations with excessive water binding (like SPI) may produce analogs with a firm texture but potentially dry mouthfeel. Taken together, combining SPI with Ory (Ory-A) enhances water-binding in chicken analogs, while SPI-alone and SPI-Leu exhibit extreme water mobility. NMR reveals how protein-ligand influences water distribution at a molecular level, guiding tailored inks for improved textural attributes.

### 3.3 Rheological and textural characters of SPI-lipo-lipids 3D-chicken analogs stabilized with gums

**Fig. 5:**
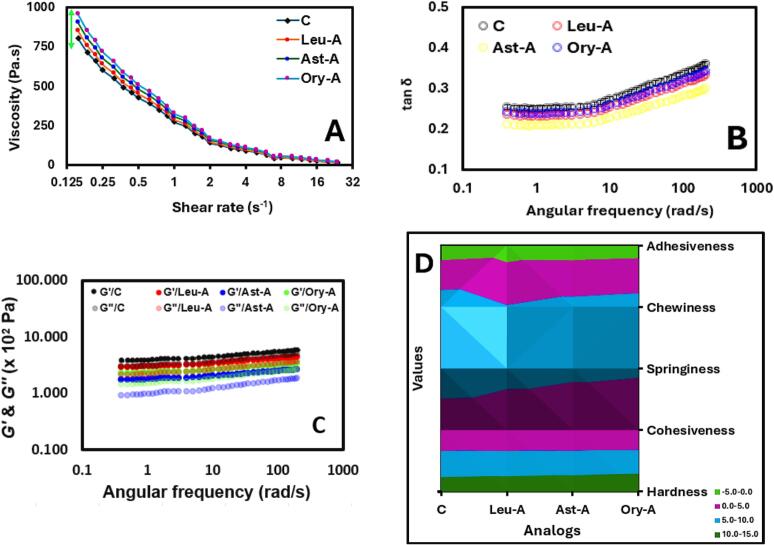
The rheological properties of each chicken analog formula ink prepared by SPI-lipo-lipids stabilized with Gellan gums. Changes in the viscosity as a function of shear rate (A), Tan as a function of angular frequency (B), and both modules as a function of angular frequency (C). The textural parameters of the printed chicken analogs after 3D-printing include hardness, cohesiveness, chewiness, adhesiveness, and springiness values (D).

The development of chicken analogs utilizing SPI and lipophilic phytonutrients represents a significant advancement in plant-based analogs. All formulations exhibit the usual shear-thinning (pseudoplastic) behavior seen by the rheological data in Fig. 5**A**, where the viscosity decreases as the shear rate increases. For extrusion-based food manufacturing techniques, which are commonly employed in the creation of chicken analogs, this characteristic is crucial. The initial viscosity of the SPI-Ory combination is greater (∼900 Pa·s) than that of SPI-Leu and SPI-Ast combinations with values of ∼800 and ∼ 850 Pa·s, respectively. This implies that when SPI is at rest, oryzanol produces a more resilient network structure. All analogs converge toward similarly low viscosities at greater shear rates (>8 s^−1^), suggesting similar flow behavior under processing circumstances involving high shear forces. There are notable variations in viscoelastic characteristics between formulations, as seen by the tan δ values (G“/G' ratio). The dynamic oscillatory rheology data further confirms these observations, with G' exceeding G" moduli across all frequencies (Fig. 5**B**), indicating the predominance of elastic behavior in these systems. Interestingly, SPI-Ory analogs show intermediate tan δ values (G"/G' ratio), closely resembling the viscoelastic qualities of actual chicken meat by achieving an ideal compromise between viscous and elastic properties (McClements, 2023). This is consistent with research showing that certain viscoelastic characteristics are essential for producing fibrous structures that resemble meat in plant-based analogs (Schreuders et al., 2022). Meanwhile, SPI-Leu showed intermediate tan δ values, whereas SPI-Ast demonstrated the lowest tan δ values, suggesting elastic behavior (Bashash, Wang-Pruski, He, & Sun, 2024). These findings are further supported by the dynamic oscillatory rheology data shown in Fig. 5**C**, where G' and G" moduli are plotted against angular frequency. The highest moduli values overall are found in SPI, SPI-Leu, and SPI-Ory, suggesting stronger network formation in those systems, while the lowest moduli values are found in the SPI-Ast, indicating weaker structural integrity. Interestingly, all formulations exhibit greater G' than G" across the entire frequency range tested, confirming the predominance of elastic behavior in these systems (Seetapan et al., 2023).

The texture characteristics that are related to sensory qualities and possible resemblances to real chicken flesh are depicted in Fig. 5**D**. Significant differences exist in the hardness values among formulations; SPI-Ory analog exhibits hardness values that are most like those seen in the literature for actual chicken meat (∼7–9 N) (Godschalk-Broers et al., 2022). SPI-Ory has better binding strength, which may improve structural integrity during processing and consumption. Cohesiveness follows a clear pattern. SPI-Ory additionally optimizes springiness values, a crucial characteristic for simulating the elasticity of meat fibers, which show textural recovery following initial compression. The improved texture of SPI-Ory analogs is consistent with research showing that balanced noncovalent protein-ligand interactions, such as hydrophobic and H-bonds, are responsible for the right texture in chicken analogs. Curiously, SPI and SPI-Ory have the highest chewiness values, although their adhesiveness is lower. This might provide benefits in processing and mouthfeel by decreasing stickiness, which is consistent with their higher cohesiveness and hardness. Ory exhibits superior interaction with SPI in GG-stabilized systems, producing chicken analogs with better structural integrity and viscoelastic properties that closely resemble real chicken meat. Overall, its distinct chemical structure allows for multiple interaction points with SPI, forming a more stable protein network.

### 3.4 Microstructural visualization of 3D-printed SPI-based multi-ligands chicken analogs

**Fig. 6:**
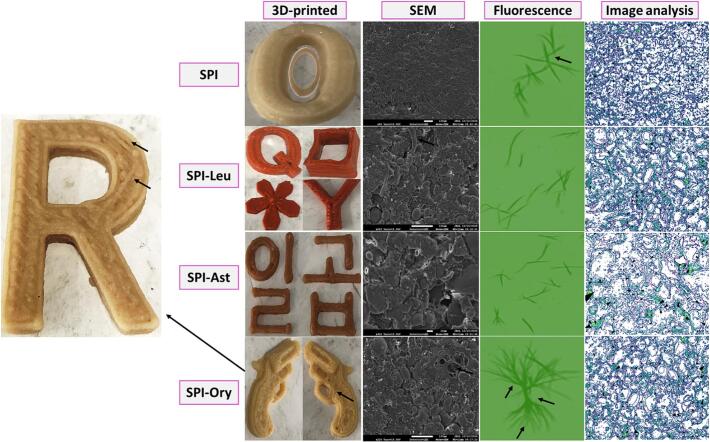
The 3D-printed, SEM snaps, and fluorescence microscopy snaps of chicken analog samples based on SPI alone or after interacting with each of lutein (Leu), astaxanthin (Ast), and γ-oryzanol (Ory), which were taken immediately after printing. All analogs contain gellan gums. The black arrows refer to the coherence and consistency in the analog structure. SEM-image plots were also done.

The integration of lipophilic phytonutrients with SPI demonstrably advances the design of functional plant-based chicken analogs by directly influencing their structural and textural properties. Comparative analysis of the 3D-printed analogs reveals that the nature of the incorporated lipo-ligand critically determines macrostructure and performance with a lipo-ligand dependent manner (Fig. 6). SPI-Ory analogs consistently outperform those containing Leu and/or Ast, exhibiting superior dimensional stability, smooth edges, and enhanced structural coherence. This can be mechanistically attributed to γ-oryzanol's molecular configuration, which promotes robust hydrophobic and H-bonds interactions with SPI, thereby reinforcing the protein matrix (Sobhy et al., 2020). In contrast, SPI-Leu and SPI-Ast analogs display moderate to poor network integrity, with more discontinuities and irregularities in both macro- and microstructure in the printed patterns. The superior performance of oryzanol aligns with findings that the selection of appropriate lipophilic compounds can enhance printability while maintaining nutritional benefits. These findings highlight the decisive role of specific lipo-ligands in modulating protein network assembly during 3D printing, underscoring the potential for targeted molecular design in plant-based meat analog development.

At the microstructural level, SEM and fluorescence imaging further substantiate these trends (Fig. 6). SPI-Ory analogs form a densely packed, fibrous network with minimal voids and uniform alignment, correlating with intense, well-organized fluorescence signals that indicate strong and homogeneous binding of the bioactive compound within the protein matrix. In contrast, SPI-Leu and SPI-Ast analogs exhibit less organized, more porous networks with weaker and more diffuse fluorescence, reflecting less effective integration, especially with SPI-Leu that shows a moderately discontinuous protein aggregation and intermediate fiber formation. SPI-Ast specimens exhibit a more irregular microstructure with larger voids and less uniform protein distribution. These microstructural differences translate directly into textural attributes relevant to consumer acceptance, with the SPI-Ory system most closely mimicking the fibrous texture of chicken meat. The more cohesive microstructure observed in SPI-Ory samples suggests stronger protein-phytonutrients interactions that enhance the fibrous network formation necessary for chicken-like textures. Likewise, SPI-Leu analogs display moderate fluorescence intensity with scattered distribution patterns, suggesting moderate interaction with SPI-matrix. Most remarkably, SPI-Ory analogs exhibit intense, well-defined fluorescence patterns with distinctive fibrous structures (indicated by black arrows), demonstrating superior binding affinity and more uniform distribution throughout the protein network. SPI-Ast analogs also show weaker and more diffuse fluorescence signals, indicating less effective binding with SPI-structure. The clearer fluorescence patterns in SPI-Ory analogs suggest more effective noncovalent binding, which explains the enhanced structural stability observed in both the 3D-printed and SEM analyses. Taken together, γ-Ory outperforms Leu and Ast in SPI-based chicken analogs, enhancing structural integrity (*via* 3D-printing), fibrous organization (SEM), and binding (fluorescence microscopy), where GG stabilizers strengthen this by forming fibrous protein networks under heat/shear. Collectively, this work demonstrates that rational selection of lipo-ligands, particularly γ-Ory, is critical for optimizing both the structure and functionality of plant-based chicken analogs, offering a clear pathway for future innovation in this field.

### 3.5 Thermal stability analysis of 3D-printed SPI-based chicken analogs

**Fig. 7:**
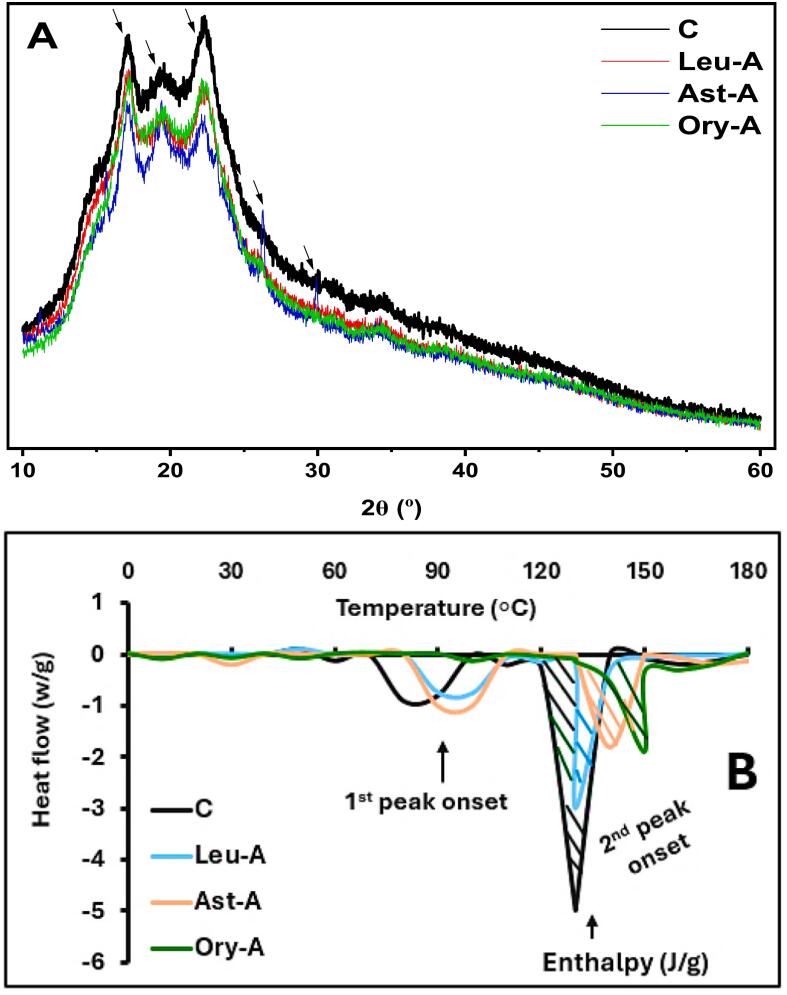
The XRD analysis (A) and DSC analysis (B) of each chicken analog sample prepared with SPI-lipo-ligands stabilized with Gellan gums.

Crucial information on the structure-function relationships of SPI and SPI-lipo-ligand based chicken analog formulations are revealed by the examination of XRD patterns and DSC thermograms (Figs. 7A-B). Each analog formula crystalline structure varies noticeably, according to the XRD patterns. With differences in peak strength, sharpness, and shoulder forms, all analogs display distinctive protein crystalline peaks in the 2θ = 18–24° range (Fig. 7A). SPI-Ory and SPI-Leu showed a comparable crystalline pattern with minor variations in peak height and width, while SPI-Ast exhibits the least pronounced peaks among all samples, suggesting lower crystalline. Meanwhile, some small new peaks were noted in the case of SPI-Ast at ∼2θ = 26–29°. SPI (C) showed the most pronounced peaks in the region around 2θ = 18–24°, suggesting a higher degree of crystallinity compared to other formulations. This region decreased, due to the potential binding between SPI and each lipo-ligand in the presence of GG. This interaction might potentially be responsible for the enhanced structural properties (Li et al., 2023) and then the thermal stability of the prepared 3D-printed chicken analogs. The crystalline features influenced the mechanical parameters necessary for successful 3D printing applications. Although it may come at the price of flexibility, more crystallinity, as shown in SPI alone, often corresponds with enhanced hardness and structural integrity.

Meanwhile, SPI-Ory and SPI-Ast exhibit intermediate crystallinity, indicating a balanced profile that might provide benefits for both printing flexibility and structural stability (Shahbazi et al., 2022). However, DSC thermograms offer vital information about the various chicken analog formulations' thermal stability (Fig. 7B). The control ink (C) exhibits two prominent endothermic transitions. The 1st peak onset occurs at ∼88 °C with moderate enthalpy change, while the 2nd peak onset appears near 125 °C with significantly greater enthalpy. This biphasic denaturation pattern is characteristic of soy protein isolate, which contains both 7S (β-conglycinin) and 11S (glycinin) globulins that denature at different temperatures. This aligns with recent findings (Burghardt et al., 2024), who identified the linear temperature processing range for soy protein to be 87–116 °C. Meanwhile, SPI-Ast displays moderately reduced enthalpy and slightly shifted transition temperatures, indicating that Ast interacts with SPI through hydrophobic interactions (agreeing with our docking results), potentially stabilizing the protein structure.

Furthermore, SPI-Ory-based analog exhibits the most distinctive thermogram, with significantly shifted and attenuated peaks, suggesting that Ory forms stronger interactions with SPI, through multiple binding mechanisms, including hydrophobic interactions and H-bonding. SPI-Leu shows intermediate effects. These varied thermal behaviors reflect differential binding affinities and mechanisms between lipo-lipids and SPI, which directly influence the structural and functional properties of the chicken analogs. Varying enthalpy values indicate differences in protein-ligand binding strength, affecting structural stability. Bioactive-enriched analogs, particularly SPI-Ory, show reduced enthalpy, implying lipophilic compounds stabilize proteins *via* hydrophobic interactions, lowering energy needs. GG-stabilized matrices enhance the texture and stability of plant-based chicken analogs by retaining lipo-lipids.

## 4 Conclusion

This study significantly advances 3D-printed chicken analog development by revealing how SPI interact with lipophilic phytonutrients and GG across four formulations. Our comprehensive analyses established that protein-ligand interactions, particularly those involving γ-oryzanol, fundamentally enhance printability and structural integrity in the final products. Multispectral analysis and molecular docking confirmed that SPI-Ory combinations form strong noncovalent interactions through H-bonding and hydrophobic forces with GG, directly strengthening ink cohesion and controlling water mobility throughout the matrix. These optimized formulations demonstrated ideal viscoelastic properties for extrusion printing while maintaining exceptional shape fidelity post-processing. Thermal analyses through DSC and XRD validated the enhanced stability of the SPI-Ory system, which directly correlated with superior layer adhesion in printed structures. In contrast, we found that single-protein formulations created weaker networks and yielded inconsistent printing results. Our findings underscore the strategic importance of engineered protein-multi-lipo-ligand interactions in plant-based chicken alternatives, with SPI-Ory systems emerging as particularly effective for creating structurally coherent chicken analogs. This work delivers practical advances for sustainable food technology by optimizing molecular-level interactions for commercial-scale 3D-food printing applications. Future research should evaluate the sensory properties of these analogs, particularly among populations with specific dietary requirements or texture preferences, to further validate their market potential.

## CRediT authorship contribution statement

**Remah Sobhy:** Writing – original draft, Methodology, Formal analysis, Conceptualization. **Ke Zhang:** Methodology, Formal analysis, Data curation. **Jianing Zhang:** Visualization, Validation, Investigation. **Mohammed Alrugaibah:** Writing – original draft, Validation, Visualization. **Thamer Aljutaily:** Writing – review & editing, Writing – original draft, Validation. **Raed Alayouni:** Writing – review & editing, Writing – original draft, Visualization. **Hassan Barakat:** Writing – review & editing, Writing – original draft, Software. **Ibrahim Khalifa:** Writing – review & editing, Writing – original draft, Resources, Project administration, Conceptualization. **Xiaobo Zou:** Writing – review & editing, Writing – original draft, Project administration, Funding acquisition, Supervision.

## Declaration of competing interest

The authors declare that they have no known competing financial interests or personal relationships that could have appeared to influence the work reported in this paper.

## Data Availability

Data will be made available on request.
